# Frizzled-related proteins 4 (SFRP4) rs1802073G allele predicts the elevated serum lipid levels during acitretin treatment in psoriatic patients from Hunan, China

**DOI:** 10.7717/peerj.4637

**Published:** 2018-04-13

**Authors:** Xingchen Zhou, Wu Zhu, Minxue Shen, Yijing He, Cong Peng, Yehong Kuang, Juan Su, Shuang Zhao, Xiang Chen, Wangqing Chen

**Affiliations:** 1 Department of Clinical Pharmacology, Xiangya Hospital, Central South University, Changsha, China; 2 Department of Dermatology, Xiangya Hospital, Central South University, Changsha, China; 3 Hunan Key Laboratory of Skin Cancer and Psoriasis, Changsha, China

**Keywords:** SFRP4, Dyslipidemia, Polymorphism, Psoriasis, Acitretin, Pharmacogenetics

## Abstract

**Background:**

Acitretin is a second-generation synthetic retinoid, and is widely used for treating the severe psoriasis vulgaris. However, it should be chosen with caution for its cardiovascular risk, and it is reported that acitretin may increase the serum lipids. The purpose of this study is to investigate the relationship between the Frizzled-related proteins 4 (SFRP4) rs1802073 polymorphism and the changes of serum lipids in Chinese psoriatic patients during the treatment with acitretin.

**Methods:**

In our study, 100 psoriatic patients were recruited systematically treated with acitretin (30 mg/day) for at least eight weeks. Data of the patients’ demographic and clinical characteristics and the results of serum triglyceride (TG), total cholesterol (TC), high-density lipoprotein cholesterol (HDL-C) and low-density lipoprotein cholesterol (LDL-C) were collected pre- and post-treatment.

**Results:**

A total of 84 psoriatic patients were enrolled and divided into three groups by SFRP4 rs1802073 genotypes. The patients who carried with TT genotype had maintained levels of TG and LDL-C after acitretin treatment, while patients with GG/GT genotypes had significantly elevated levels of serum TG and LDL-C compared to the TT genotype (ΔTG%: 27.53 ± 59.13 vs −1.47 ± 37.79, *p* = 0.026, ΔLDL-C%: 10.62 ± 26.57 vs −1.29 ± 17.07, *p* = 0.042). The association of rs1802073 with TG and LDL-C profiles remained significant after adjusting for age, gender, and body mass index. Although without significance, the pre-post change in serum level of TC across rs1802073 GG/GT genotypes demonstrated a trend similar to TG and LDL, and the serum level of HDL-C demonstrated a trend opposite to TG, TC and LDL.

**Conclusions:**

Our results demonstrated that SFRP4 rs1802073 polymorphism was found to be associated with elevated serum lipid levels after acitretin treatment, and it may serve as a genetic marker of safe and precise treatment for individual psoriatic patients.

## Introduction

Psoriasis is an immunologically mediated chronic inflammatory skin disorder that affects 2%–3% of the general population (Boehncke & Schon, 2015; Grayson, 2012; Griffiths & Barker, 2007). The full pathogenesis of the disease is still not completely understood, which might include genetic (Qi et al., 2014; Wu et al., 2011) immunological (Bowcock, 2005; Liu et al., 2017) and abnormal metabolism (Asefi et al., 2012; Zhao et al., 2013). Acitretin is a second generation synthetic retinoid and widely used for moderate to severe psoriasis vulgaris (Katz, Waalen & Leach, 1999; Ling, 1999), while the mechanism of pharmacology is unclear. Acitretin is thought to function through regulating the differentiation and proliferation of epidermal keratinocytes, and impacting on Th1 and Th17 cells’ number and function (Dogra & Yadav, 2014; Niu et al., 2012).

Although acitretin is a common systemic agent for treating psoriasis in clinical, the efficacy of acitretin is notoriously variable. As reported, the response rate of acitretin was 46%–52% after 12 weeks (Ormerod, Campalani & Goodfield, 2010). Some dose-ranging researches suggested that there was a dose–response trend of response, with the highest doses of acitretin (50–75 mg/day) proving more effective than lower doses (10–25 mg/day) (Goldfarb et al., 1988; Lassus et al., 1987). The hyperlipidemia is an obvious side-effect of acitretin, particularly hypertriglyceridemia (Orfanos et al., 1997). The higher dosage increasing the triglyceride levels occurred in 66% of psoriatic patients and total cholesterol occurred in 33% (Katz, Waalen & Leach, 1999). Thus, it should be chosen with caution for its possible elevated risks of serum lipid profile and cardiovascular events (Balta et al., 2013; Orfanos et al., 1997). This limits its clinical use, especially in patients with other risk factors for cardiovascular diseases. Therefore, it is necessary to identify a genetic marker to predict the elevated serum lipid levels during acitretin treatment.

Until now, there were few pharmacogenetics studies that focused on acitretin. Campalani and colleagues found that there was an association between the apolipoprotein E gene (APOE) e4 (+3937C/+4075C) alleles and psoriasis, and demonstrated there was a 10% e4 allele frequencies increased in those patients with drug-induced hypertriglyceridemia; however, it is not statistically significant in their research (Campalani et al., 2006).

In our previous research, we showed that secreted frizzled-related proteins 4 (SFRP4) rs1802073G>T was a missense mutation and was significantly associated with the response to acitretin (Zhou et al., 2017). SFRP4 is a frizzled decoy receptor that binds to Wnt/β-catenin and inhibits the Wnt/β-catenin signaling pathway, through competing surface receptors (Carmon & Loose, 2008). Furthermore, it has been reported that the Wnt pathway might be associated with raised the serum levels of cholesterol, triglycerides, and low-density lipoprotein through low-density lipoprotein receptor-related protein5/6 (LRP5/6) (Go & Mani, 2012), which regulates the clearance of LDL-C and TG, and involved in synthesis of TG and fatty acids (Liu et al., 2008; Tomaszewski et al., 2009). Therefore, SFRP4 may influence the lipid metabolism by Wnt signaling pathway.

The purpose of this study was to investigate the relationship between SFRP4 rs1802073 and the changes of serum lipids in Chinese psoriatic patients from Hunan province during the treatment with acitretin, and expected to determine whether it can be as a genetic marker to predict the changes in serum lipids.

## Materials and Methods

### Patients

**Figure 1:**
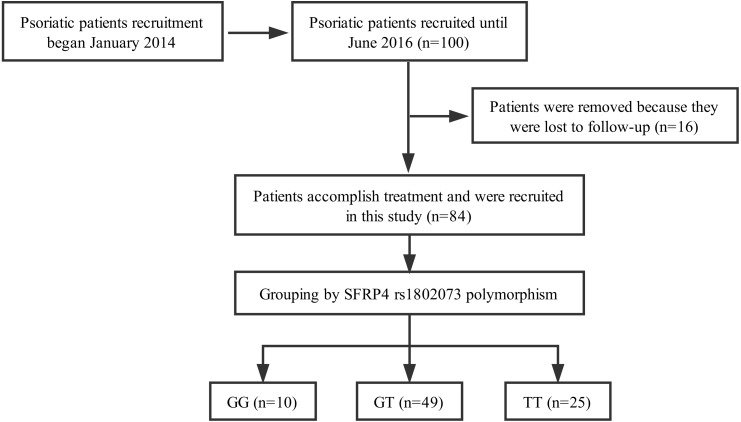
Flow diagram: acitretin was prescribed and provided for all subjects at the time of enrollment. A total of 84 patients accomplished at least 8 weeks treatment. Sixteen patients were removed because of lack of follow-up.

From January 2014 to June 2016, a total of 100 Han Chinese patients with psoriasis vulgaris from Hunan province were recruited through Xiangya Hospital, Central South University (Changsha, China). The severity and extensiveness of the disease was assessed by Psoriasis Area and Severity Index (PASI) score, and all patients collected in our study were diagnosed as moderate-to-severe state (PASI score ≥ 10). This research was approved by the Ethic Committee of Xiangya Hospital, and the clinical trial registration numbers were ChiCTR-OCH-14004518 (Chinese Clinical Trial Registry online) and NCT02715960 (ClinicalTrials.gov). The study was conducted in accordance with the Declaration of Helsinki. Informed written consents have been obtained from every patient. All patients were systematically treated with acitretin (30 mg/day) for at least eight weeks. In total, 84 patients completed the study, while 16 patients were removed because of lost to follow-up (Fig. 1). Data of the patients’ demographic and clinical characteristics and the results of serum triglyceride (TG), total cholesterol (TC), high-density lipoprotein cholesterol (HDL-C) and low-density lipoprotein cholesterol (LDL-C) were collected.

### DNA extraction and sequencing

The genomic DNA was extracted by a commercial DNA extraction kit (QIAamp; Qiagen, Hilden, Germany) from the venous blood samples of all patients and stored at −80 °C until used. The purity and concentration of all the samples were determined by the Bio-spec Nano Spectrophotometer. Genotyping for SFRP4 rs1802073 polymorphism was analyzed by the Sequenom MassARRAY system (Sequenom, San Diego, CA, USA) (Gabriel, Ziaugra & Tabbaa, 2009).

### Statistical analysis

The primary outcome of this study was the relative change in lipid profile after acitretin treatment, and was calculated as (post-treatment lipid level − pre-treatment lipid level)/pre-treatment lipid level × 100%. Continuous data were described as mean ± standard deviation (SD), and were tested using one-way ANOVA. Categorical data were presented as numbers (%), and were compared using a chi-square test. A chi-square test was also to determine whether genotypes distribution of SFRP4 rs1802073 polymorphism agreed with Hardy–Weinberg equilibrium. The results were adjusted for gender, age, and BMI using multiple linear regression model. Beta and 95% confidence intervals (CI) were estimated to show the effect size of the genotypes on the pre-post change in levels of TG, TC, HDL-C and LDL-C changed in patients who carry certain haplotypes. A two-tailed *p* value < 0.05 was regarded to be statistically significant. All the analyses were performed in the SPSS software version 18.0 (IBM corporation, Armonk, NY, USA).

## Results

### Patient characteristics

A total of 84 psoriatic patients with moderate to severe psoriasis vulgaris were collected in this study. Ten patients carried with rs1802073 GG genotype, 49 patients carried with rs1802073 GT genotype and 25 patients carried with rs1802073 TT genotype. The characteristics of these patients including age, body mass index (BMI), sex, are shown in Table 1. There were no significant differences in age, gender and BMI across different SFRP4 rs1802073 genotypes. The serum levels of TG, TC, HDL-C and LDL-C before treatment were also not associated with the genotypes.

**Table 1:** Characteristics of the patients in relation to the rs1802073 genotypes.

	GG	GT	TT	*p*
	(*n* = 10)	(*n* = 49)	(*n* = 25)	
Age, y	46.80 ± 11.96	37.84 ± 11.40	41.76 ± 15.69	0.105
BMI, kg∕m^2^	23.36 ± 5.51	22.97 ± 3.32	22.61 ± 4.55	0.868
TG (mmol/l)^a^	1.33 ± 0.61	1.35 ± 0.58	1.57 ± 1.09	0.477
TC (mmol/l)^a^	4.81 ± 0.71	4.78 ± 0.92	4.97 ± 1.02	0.72
LDL-C (mmol/l)^a^	2.56 ± 0.63	2.69 ± 0.73	2.89 ± 0.92	0.442
Male	6 (60.0%)	40 (81.6%)	15 (60.0%)	0.09
Female	4 (40.0%)	9 (18.4%)	10 (40.0%)	

**Notes.**

BMI body mass index TG triglyceride TC total cholesterol LDL-C low-density lipoprotein cholesterol

a TG, TC and LDL-C were pre-treatment measurements.

### Changes of serum lipid profiles between pre- and post-therapy

We determined the serum levels of TG, TC, HDL-C and LDL-C in 84 psoriatic patients. When compared to the baseline, the serum level of TG was significantly elevated (1.41 ± 0.77 *vs* 1.62 ± 1.05 mmo/L, *p* = 0.009) and the serum level of HDL-C was significantly decrease (1.42 ± 0.30 *vs* 1.35 ± 0.27 mmo/L, *p* = 0.001), while the serum levels of TC and LDL-C were also elevated but had no significant differences (Table 2).

**Table 2:** Comparison on lipid parameters of patients between pre-/post-therapy groups.

	Pre-therapy	Post-therapy	Difference	Percent change^a^	*p*^b^
TG (mmol/l)	1.41 ± 0.77	1.62 ± 1.05	0.21 ± 0.73	18.90 ± 55.08	**0.009**
TC (mmol/l)	4.84 ± 0.93	4.89 ± 0.89	0.05 ± 0.70	2.36 ± 16.13	0.503
HDL-C (mmol/l)	1.42 ± 0.30	1.35 ± 0.27	−0.07 ± 0.20	−4.02 ± 13.03	**0.001**
LDL-C (mmol/l)	2.74 ± 0.79	2.85 ± 0.73	0.11 ± 0.60	7.08 ± 24.65	0.100

**Notes.**

a Percent change was calculated as (pre-post difference/baseline) × 100%, indicating the relative change between pre- and post-treatment measurements.

b *p* value derived from the statistical tests for pre-post differences, using paired *t* tests.

### SFRP4 rs1802073 polymorphism was associated with the pre-post change in serum levels of TG and LDL-C

We analyzed the influence of SFRP4 rs1802073 polymorphism on the serum levels of TG, TC, HDL-C and LDL-C between the pre and post-therapy of the 84 psoriatic patients. Genotype distribution of this polymorphism was in Hardy-Weinberg equilibrium (*p* > 0.05). We found that SFRP4 rs1802073 polymorphism were significantly associated with the percent change of serum levels of TG and LDL-C in the three genotypes (GG/GT/TT). The patients who carried with TT genotype had maintained levels of serum TG and LDL-C, while patients with GG/GT genotypes had significantly elevated levels of serum TG and LDL-C compared to the TT genotype (ΔTG%: 27.53 ± 59.13 vs −1.47 ± 37.79, *p* = 0.026, ΔLDL-C%: 10.62 ± 26.57 vs −1.29 ± 17.07, *p* = 0.042). Furthermore, a dose–response relationship was observed in pre-post percent change of LDL-C across GG/GT genotypes. Although without significance, the pre-post change in serum level of TC across rs1802073 GG/GT genotypes demonstrated a trend similar to TG and LDL, and the serum level of HDL-C demonstrated a trend opposite to TG, TC and LDL (Table 3, Table S1).

**Table 3:** Association of rs1802073 genotypes with the pre-post percent change of TG, TC and LDL-C.

	ΔTG%		ΔTC%		ΔHDL-C%		ΔLDL-C%	
	Mean	*p*^a^	Mean	*p*^a^	Mean	*p*^a^	Mean	*p*^a^
TT	−1.47 ± 37.79		−0.50 ± 13.05		−3.22 ± 13.05		−1.29 ± 17.07	
GT	31.08 ± 63.37	**0.016**	2.88 ± 17.05	0.397	−3.99 ± 12.28	0.812	9.04 ± 26.95	0.086
GG	10.14 ± 26.68	0.565	6.95 ± 18.64	0.221	−6.11 ± 17.36	0.560	18.37 ± 24.43	**0.032**
GG + GT	27.53 ± 59.13	**0.026**	3.57 ± 17.23	0.292	−4.35 ± 13.12	0.719	10.62 ± 26.57	**0.042**

**Notes.**

a Using one-way ANOVA and independent sample *t*-test; Δ% means percent change, i.e., the relative change between pre- and post-therapy measurements.

### Association of SFRP4 rs1802073 polymorphism with serum levels of TG and LDL-C adjusted for confounders

Multiple linear regression models demonstrated that compared to the TT genotype, the psoriatic patients who carried GG/GT had significantly elevated levels of serum TG and LDL-C (ΔTG%: *β* = 26.92 [1.17, 52.67], *p* = 0.041; ΔLDL-C%: *β* = 12.27 [0.61, 23.93], *p* = 0.039). The relationship between the change in serum level of TC and HDL-C and the three rs1802073 genotypes remained non-significant after adjusting for age, gender and BMI (Table 4, Table S2).

**Table 4:** Association of rs1802073 genotypes with the percent change of TG, TC and LDL-C via univariate general linear model.

	ΔTG%		ΔTC%		ΔHDL-C%		ΔLDL-C%	
	Beta [95% CI]^b^	*p*^a^	Beta [95% CI]^b^	*p*^a^	Beta [95% CI]^b^	*P*^a^	Beta [95% CI]^b^	*p*^a^
TT	Ref		Ref		Ref		Ref	
GT	31.86 [5.10, 58.63]	**0.020**	3.51 [−4.53, 11.55]	0.387	−1.91 [−8.45, 4.63]	0.562	10.75 [−1.44, 22.94]	0.083
GG	7.43 [−32.17, 47.04]	0.710	5.92 [−5.98, 17.82]	0.325	−3.26 [−12.94, 6.42]	0.505	18.25 [0.21, 36.28]	**0.047**
GG + GT	26.92 [1.17, 52.67]	**0.041**	4.00[−3.67, 11.66]	0.302	−2.19[−8.42, 4.04]	0.487	12.27[0.61, 23.93]	**0.039**

**Notes.**

a Adjusted for age, gender and body mass index (BMI).

b Beta is the partial regression coefficient of the genotypes; it indicates the between-group difference in pre-post percent change in lipid profile in psoriatic patients who received acitretin treatment. Beta can be interpreted as, for example, the percent change in serum level of triglyceride among patients with GG/GT genotypes was 26.92% greater than that among patients with TT genotype.

## Discussion

In this study, we identified a significant association of SFRP4 rs1802073 polymorphism with elevated serum lipid profile in psoriatic patients after the treatment with acitretin. Our results demonstrated that the psoriatic patients who carried GG/GT genotype had significantly elevated serum levels of TG and LDL-C after receiving acitretin. Furthermore, a dose–response relationship was observed in pre-post percent change of LDL-C across the three genotypes. Although the trend of change in TC across the GG/GT/TT genotype was consistent with that in TG and LDL-C, the result was not statistically significant.

Acitretin is a retinoid analog belonging to the family of retinoid, and is also thought to be a member of the RAR agonist family. SFRP4 contains a cysteine-rich domain homologous to the putative Wnt binding site of frizzled proteins, and acts as a negative regulator of Wnt signaling (Carmon & Loose, 2008). The expression of SFRP4 was upregulated by retinoic acid receptor (RAR) agonist (Green et al., 2017), hence we speculated that acitretin may alter the expression of SFRP4, such as encoding soluble Wnt signaling antagonists (Walsh & Andrews, 2003). CCAAT/enhance-binding protein-*α* (CEBPA) and peroxisome proliferator-activated receptor-*γ* (PPARγ) were regarded as the transcription factors for mastering adipogenic, blocking the induction of them and led to the represses adipogenesis through the Wnt signaling (Ross et al., 2000).

LRP5/6 play a pivotal role in cholesterol homeostasis and lipid metabolism (Borrell-Pages, Carolina Romero & Badimon, 2015; Foer et al., 2017), and act as co-receptor of the canonical Wnt/β-catenin pathway (Mi & Johnson, 2005). In addition, pravastatin (a member of the drug class of statins) is used for the treatment of dyslipidemia and the prevention of steroid-induced osteonecrosis of the femoral head (ONFH) by activating Wnt signaling pathway, include increasing the expression of LRP5, β-catenin and suppressing the expression of PPARγ (Jiang et al., 2014; Nozaki et al., 2012). Therefore, it is certainly believed that the Wnt signaling pathway is known to play a major role in adipogenesis and lipid metabolism.

The genetic variants in SFRP4 gene may affect the functions of Wnt signaling pathway and showed a relationship with diseases. A previous study compared the genotypes distribution between renal cell carcinoma (RCC) patients and controls showed that SFRP4 rs1802074 polymorphism was related to RCC susceptibility. When compared with the GG/AG genotypes, the AA genotype was found to had a marginal significance to increase renal cancer risk (Hirata et al., 2009). Meanwhile, SFRP4 c1019G9A polymorphism has been reported to be associated with the bone mineral density (BMD), and the AA genotype had a higher level of serum bone alkaline phosphatase and lower lumbar spine BMD when compared with the GG genotype, suggesting a higher risk for osteoporosis at the lumbar spine was found in patients who carriers of AA genotype (Lee et al., 2010).

In our previous study, we found SFRP4 rs1802073 polymorphism was associated with the response to acitretin, namely, the psoriatic patients who carries T allele had a better response than that in G allele carriers (Zhou et al., 2017). SFRP4 rs1802073 polymorphism is a missense mutation (G>T), and induce a proline to change into threonine at position 320 in the SFRP4 protein. SFRP4 rs1802073 (Pro320Thr) was judged to be “possibly damaging” by PolyPhen (a computer program) (Hirata et al., 2009). In this study, we confirmed that it may influence the lipid metabolism during acitretin treatment.

As we know, the moderate-to-severe psoriasis patients is required an individualized approach during a long-term treatment with acitretin, because psoriasis patients are more susceptible to vascular diseases (Asefi et al., 2012; Houshang et al., 2014). It is necessary to select a treatment based on the clinical presentation of psoriasis and contraindications. Meanwhile, the genetic marker may provide some useful reference.

We investigated the association between the SFRP4 rs1802073 polymorphism and the changes of serum levels of TG, TC, HDL-C and LDL-C for the first time. However, there are several limitations of this study, such as limited samples and single center. We hope our results can provide some useful references for further investigations into the mechanism of elevated serum lipid levels after acitretin treatment.

## Conclusions

SFRP4 rs1802073 GG/GT genotypes were found to be associated with elevated serum lipid levels after acitretin treatment. This may explain dyslipidemia observed in some of the Chinese psoriatic patients from Hunan province treated with acitretin. Our results demonstrated that SFRP4 rs1802073 polymorphism may serve as a genetic marker to predict the elevated serum lipid profile as a side-effect of acitretin, guiding individual patients for safe and precise treatment, and minimizing unnecessary expenditure.

##  Supplemental Information

10.7717/peerj.4637/supp-1 Table S1 Association of rs1802073 genotypes with the pre-post difference of TG, TC and LDL-C (1) Using one-way ANOVA; Δ means the between-group difference in lipid profile in psoriatic patients, i.e., the change between pre- and post-therapy.

10.7717/peerj.4637/supp-2 Table S2 Association of rs1802073 genotypes with the difference of TG, TC and LDL-C via univariate general linear model (1) Adjusted for age, gender and body mass index (BMI). (2) Beta is the partial regression coefficient of the genotypes; it indicates the between-group difference in lipid profile in psoriatic patients who received acitretin treatment. Beta can be interpreted as, for example, the difference in serum level of LDL among patients with GG/GT genotypes was 0.34 greater than that among patients with TT genotype.

10.7717/peerj.4637/supp-3 Data S1 Raw data

## Additional Information and Declarations

### Data Availability

The following information was supplied regarding data availability: Raw data is available in the Supplemental Information.
