# Supplementary material for: Frizzled-related proteins 4 (SFRP4) rs1802073G allele predicts the elevated serum lipid levels during acitretin treatment in psoriatic patients from Hunan, China
Source: PeerJ. 2018 Apr 13;6:e4637. doi: 10.7717/peerj.4637 (PMC5900929; doi:10.7717/peerj.4637)
Supplement: Table S1 — (1) Using one-way ANOVA; Δ means the between-group difference in lipid profile in psoriatic patients, i.e., the change between pre- and post-therapy. [file peerj-06-4637-s001.docx]

|  | △TG | | △TC | | △HDL-C | | △LDL-C | | |
| --- | --- | --- | --- | --- | --- | --- | --- | --- | --- |
|  | Mean | *p*^1^ | Mean | *p*^1^ | Mean | *p*^1^ | Mean | | *p*^1^ |
| TT | -0.02±0.55 |  | -0.10±0.63 |  | -0.05±0.19 |  | -0.13±0.53 |  | |
| GT | 0.34±0.81 | **0.040** | 0.07±0.71 | 0.305 | -0.07±0.19 | 0.658 | 0.16±0.61 | | **0.046** |
| GG | 0.14±0.35 | 0.551 | 0.33±0.80 | 0.104 | -0.13±0.26 | 0.266 | 0.43±0.56 | | **0.012** |
| GG+GT | 0.31±0.76 | **0.054** | 0.12±0.73 | 0.190 | -0.08±0.20 | 0.500 | 0.21±0.61 | | **0.019** |
