# Supplementary material for: Frizzled-related proteins 4 (SFRP4) rs1802073G allele predicts the elevated serum lipid levels during acitretin treatment in psoriatic patients from Hunan, China
Source: PeerJ. 2018 Apr 13;6:e4637. doi: 10.7717/peerj.4637 (PMC5900929; doi:10.7717/peerj.4637)
Supplement: Table S2 — (1) Adjusted for age, gender and body mass index (BMI). (2) Beta is the partial regression coefficient of the genotypes; it indicates the between-group difference in lipid profile in psoriatic patients who received acitretin treatment. Beta can be interpreted as, for example, the difference in serum level of LDL among patients with GG/GT genotypes was 0.34 greater than that among patients with TT genotype. [file peerj-06-4637-s002.docx]

|  | △TG | | △TC | | △HDL-C | | △LDL-C | |
| --- | --- | --- | --- | --- | --- | --- | --- | --- |
|  | Beta [95% CI]^2^ | *p*^1^ | Beta [95% CI] ^2^ | *p*^1^ | Beta [95% CI] ^2^ | *p*^1^ | Beta [95% CI] ^2^ | *p*^1^ |
| TT | Ref |  | Ref |  | Ref |  | Ref |  |
| GT | 0.40[0.04,0.75] | 0.030 | 0.18[-0.17,0.53] | 0.313 | -0.04[-0.14,0.06] | 0.445 | 0.29[-0.01,0.58] | 0.059 |
| GG | 0.10[-0.43, 0.63] | 0.711 | 0.38[-0.14, 0.90] | 0.153 | -0.09[-0.24,0.06] | 0.222 | 0.54[0.10,0.98] | 0.016 |
| GG+GT | 0.34[-0.01, 0.68] | 0.055 | 0.22[-0.12, 0.56] | 0.198 | -0.05[-0.14,0.05] | 0.308 | 0.34[0.05,0.62] | 0.021 |
